# Cytotoxicity of Different Concentrations of Silver Nanoparticles and Calcium Hydroxide for MC3T3‐E1 Preosteoblast Cell Line

**DOI:** 10.1002/cre2.70075

**Published:** 2025-02-18

**Authors:** Farzaneh Afhkami, Paniz Ahmadi, Golriz Rostami

**Affiliations:** ^1^ Tehran University of Medical Sciences Tehran Iran; ^2^ Private practice Tehran Iran; ^3^ Private practice Toronto Canada

**Keywords:** calcium hydroxide, cytotoxicity tests, dental disinfectants, silver nanoparticles, triple antibiotic Paste

## Abstract

**Introduction:**

With the advances in nanotechnology, nanomaterials are increasingly used in various fields due to their antibacterial properties; therefore, assessing the benefits and risks associated with the application of medicaments is imperative. This study evaluated the cytotoxicity of different concentrations of silver nanoparticles (AgNPs) and calcium hydroxide (CH) for MC3T3‐E1 preosteoblast cell line.

**Material and Methods:**

The MC3T3‐E1 preosteoblast cells were exposed to triple antibiotic paste (TAP), AgNPs, CH, and different concentrations of AgNPs mixed with CH in 1:1, 1:2, and 1:3 ratios for 24, 48, and 72 h. Cytotoxicity was evaluated by the methyl thiazolyl tetrazolium (MTT) assay, and also the colony formation assay (CFA) was performed.

**Results:**

At 24 h, the TAP and AgNPs groups showed the highest and the CH‐AgNPs/1:3 group had the lowest cell viability percentage in comparison to the other experimental groups. At 48 h, the TAP group showed the highest and the CH‐AgNPs/1:3 group showed the lowest cell viability. At 72 h, the AgNPs and CH groups showed the highest viability, while the lowest viability was noted in the CH‐AgNPs/1:3 and CH‐AgNPs/1:2 groups.

**Conclusion:**

AgNPs showed the least cytotoxic effects in all periods. The addition of AgNPs to CH increases the cytotoxic effects of CH on experimental cells. After 48 and 72 h, CH‐AgNPs/1:1 showed significantly higher cell viability in comparison to higher concentrations.

## 1 Introduction

Endodontic treatment of immature permanent necrotic teeth is a clinical challenge due to incomplete root development, difficult debridement, and higher risk of root fracture as a result of thin root canal walls (Latham et al. 2016; Khoshkhounejad et al. 2019). Such teeth are conventionally treated with calcium hydroxide (CH) or mineral trioxide aggregate. CH has been widely used for apexification and inducing the formation of an apical hard tissue barrier. However, none of the abovementioned methods can regenerate the injured tissue in the root canal space and stimulate root maturation through thickening of the root canal walls and induction of apical closure (Kim et al. 2018; Jain et al. 2020).

Regenerative endodontic procedures (REPs) have been recently used as a clinical treatment for necrotic open apex teeth. Induction of bleeding in REPs provides a source of stem cells that probably originate from the apical papilla and help in dentinal wall formation and promote constant root development and reinstatement of pulp function (Gougousis et al. 2019; Khoshkhounejad et al. 2019). Therefore, it is referred to as a “biologically based” treatment in endodontics (Khoshkhounejad et al. 2019). The regeneration process in endodontics is based on restoring and preserving the damaged teeth, their architectural features, and their biological functions (Jain et al. 2020). Apical stem cells participate in increasing the width of root dentinal walls and regeneration of preapical tissue (McIntyre et al. 2019; Jain et al. 2020). During this procedure, it is important to perform root canal debridement by chemical disinfection rather than mechanical instrumentation; furthermore, the safety of medicaments and their optimal antimicrobial activity are important points to consider in this procedure (Khoshkhounejad et al. 2019; Selis et al. 2019).

Calcium hydroxide (CH) is a conventional intracanal medicament applied in the root canal system to create a bacteria‐free biological environment and induce apexification in immature teeth. Triple antibiotic paste (TAP) is another widely used intracanal medicament for disinfection in REPs. It was introduced by Hoshino et al. in 1996 and is composed of ciprofloxacin, metronidazole, and minocycline (Banchs and Trope 2004; Latham et al. 2016; Selis et al. 2019). TAP can disinfect the necrotic infected pulp tissue and create an appropriate environment for vital tissue regeneration. Also, evidence shows that application of TAP and CH as intracanal medicaments during REPs brings about more positive results with respect to gaining root length than other nonsurgical root canal treatments and mineral trioxide aggregate (Bose, Nummikoski, and Hargreaves 2009; Mohammadi et al. 2018). TAP was introduced with the aim of achieving a sustained and advanced level of disinfection. However, such medicaments have cytotoxic effects on stem cells of the apical papilla (SCAPs) and dental pulp stem cells and can compromise the biological environment of the root canal system (McIntyre et al. 2019).

Nowadays, nanoparticles are developing fast and have several biomedical applications. Silver (Ag) has long been used as an antibacterial agent (even before the advent of antibiotics), and has several medical applications. It is used in bandages, wound dressings, ointments, catheters, implants, and prostheses (Mozayeni et al. 2014; Chan, Zhang, and Cheung 2015; Xavier et al. 2015). Silver nanoparticles (AgNPs) generally range in size from 1 to 100 nm (Ge et al. 2014) and contain 20–15,000 silver atoms (Ahari et al. 2011). The increased surface‐to‐volume ratio of AgNPs is responsible for their improved physical, chemical, and biological properties (Ge et al. 2014). Since the biofilm of microorganisms is resistant to antibacterial agents, the increased surface‐to‐volume ratio of AgNPs makes it possible for them to easily penetrate through the cell membrane, damage the DNA, and finally cause the death of the microorganisms (Bapat et al. 2018). It has been proven that AgNPs have the potential to kill about 650 different microorganisms, including bacteria, fungi, and viruses, and have anti‐inflammatory and regenerative effects (Bapat et al. 2018). Despite such benefits, AgNPs are known to induce cytotoxic effects on different cell types (Ge et al. 2014), such as peripheral blood mononuclear cells (Shin et al. 2007) and human alveolar macrophage cell line (Soto, Garza, and Murr 2007). However, size, shape, chemical composition, surface charge, solubility, and other features of AgNPs can affect their cytotoxicity (Beer et al. 2012).

The cytotoxicity of AgNPs is also related to the amount of silver (Ag+ ) ions in the solution, and higher concentrations can further decrease the cell viability (Beer et al. 2012). Studies have shown that AgNPs in low concentrations have no significant toxic effects on human gingival fibroblast cell line (Zorraquín‐Peña et al. 2020), human dental pulp stem cells (Dutra‐Correa et al. 2018) and L929 murine fibroblast cell line (Takamiya et al. 2016). AgNPs can cross the blood–brain barrier (Ahari et al. 2011), impair mitochondrial function (Bapat et al. 2018), and interfere with the action potential of the cell membrane (Bapat et al. 2018). On the other hand, the antibacterial effects of AgNPs have been reported in various studies (Javidi et al. 2014; Afkhami et al. 2015; Shrestha and Kishen 2016; Matoug‐Elwerfelli et al. 2021). Thus, their potential benefits and detriments should be balanced. Despite the extensive evidence available regarding the antibacterial effects of AgNPs, their cytotoxicity has not been well investigated. Due to the potential cytotoxic effects of medicaments used in root canal treatment through the apex, the present study aimed to assess the cytotoxic effects of different doses of CH and AgNPs on the viability and proliferation of MC3T3‐E1 preosteoblast cell line after different exposure times.

Despite the extensive evidence available regarding the antibacterial effects of AgNPs, their cytotoxicity has not been well investigated. Thus, the present study aimed to assess the cytotoxic effects of different doses of CH and AgNPs on the viability and proliferation of the MC3T3‐E1 preosteoblast cell line after different exposure times.

## 2 Materials and Methods

### 2.1 Cell Culture

The MC3T3‐E1 preosteoblast cell line was chosen according to previous studies (Imazato et al. 2006; Jun, Lee, and Lee 2021) and was obtained from the REIKEN BioResource Center (Saitama, Japan). MC3T3‐E1 cells were cultured in Dulbecco's modified Eagle's medium (DMEM) (Gibco Invitrogen, Grand Island, NY, USA) with 10% fetal bovine serum (Gibco, Gaithersburg, MD, USA), and antibiotics (100 U/mL penicillin and 100 µg/mL streptomycin; Gibco Invitrogen), and incubated at 37°C with 5% CO_2_ to reach approximately 100% confluence. Untreated MC3T3‐E1 cells (with medium only) served as the control group. The culture medium was refreshed every 3 days.

The cells were detached from the bottom of the cell culture flask by using 0.25% trypsin and 0.05% EDTA (Sigma‐Aldrich, St Louis, MO, USA) and were replated at a density of 1 × 10^5^ Cells/Well for 48 h to allow the cells to attach to the bottom of the wells. The cells were then treated with different medications.

### 2.2 Experimental Groups

The experimental groups were as follows:

1. Control group
2. AgNPs
3. CH
4. CH + AgNPs in 1:1 ratio (CH‐AgNPs/1:1)
5. CH + AgNPs in 1:2 ratio (CH‐AgNPs/1:2)
6. CH + AgNPs in 1:3 ratio (CH‐AgNPs/1:3)
7. TAP

Each plate was assessed at 24, 48, and 72 h after adding the medium. At each experimental time point, the culture medium was removed, and then the methyl thiazolyl tetrazolium (MTT) assay was performed. Colony formation assay (CFA) was carried out after 7 days.

### 2.3 Preparation of Medicaments

The medicaments evaluated in this study (CH, TAP, AgNPs) were prepared according to the following instructions:

1. TAP: 250 mg ciprofloxacin tablet (Aria Darou, Tehran, Iran), 250 mg metronidazole tablet (Pars Darou, Tehran, Iran), and 100 mg minocycline capsule (TeoPharma, Pavia, Italy) were used. Equal weights of the three antibiotics (1:1:1) were placed on a mixing pad and mixed with 1 mL sterilized water by a spatula to produce a concentration of 1 mg/mL
2. CH (Golchadnet, Tehran, Iran): 1 mg/mL concentration of CH was prepared.
3. To prepare AgNPs (NanoSany Corporation, Mashhad, Iran), a 100 ppm suspension was used. The mean diameter of the particles was 20 nm.
4. The combination of CH and AgNPs was prepared by mixing AgNP suspension with CH at a proportion of 1:1, 1:2, and 1:3.

### 2.4 MTT Assay

MTT method assesses the mitochondrial activity of cells. This test quantifies the conversion of water‐soluble MTT, into insoluble formazan. In the next step, the insoluble formazan is solubilized and its concentration is measured as optical density in a spectrophotometer (da Silva et al. 2020). In this study, the cell viability was assessed after 24, 48, and 72 h by the MTT colorimetric assay.

At the respective time points, the cell culture medium was removed, and 50 µL of the 3‐(4,5‐dimethylthiazol‐2‐yl)‐2,5‐diphenyltetrazolium bromide (MTT) solution (5 mg/mL; Sigma Chemical Co., St Louis, MO, USA) was added to each well. The plate was incubated for 4 h (5% CO_2_ and 98% humidity at 37°C temperature) to allow MTT reduction by the metabolically active cells. After the incubation period, the MTT solution was washed twice with phosphate‐buffered saline (PBS), and 100 µL of Dimethylsulfoxide (DMSO) (Sigma‐Aldrich) was added to each well, resulting in the dissolving of crystals in viable cells. The solution was transferred to an ELISA microplate reader (Anthos 2020, Biochrome, Cambridge, UK), and the absorbance was read at 540 nm wavelength (Yeh et al. 2009; López‐García et al. 2023) Next, the cell viability percentage was calculated by dividing the mean optical density (OD) of the respective group by the mean OD of the control group at the same time point multiplied by 100.

#### 2.4.1 Colony Formation Assay

Cytotoxicity was also determined by the colony formation assay (CFA) of MC3T3‐E1 preosteoblast cells treated with each of the medicaments. For this purpose, the cells were treated with the medicaments. After changing the culture medium with the fresh medium, cells were incubated for 7 days. After this period, cells were washed with phosphate‐buffered saline (PBS), and the colonies were fixed with 80% methanol and stained with a 3% Giemsa solution. The number of colonies with more than 50 cells was then counted, and the relative CFA was calculated as the number of colonies in the treated dishes divided by the number in control dishes and multiplied by 100 (Yeh et al. 2009; Kobayashi et al. 2013).

### 2.5 Statistical Analysis

The normality of the data was assessed using the Kolmogorov–Smirnov test, and the homogeneity of variances was analyzed using Levene's test. Two‐way ANOVA, one‐way ANOVA, and Tukey's HSD test were used to compare the CFA and MTT cell viability percentages. The experimental groups were compared with the control group at different time points using the Mann–Whitney *U* test. The experimental data were analyzed by SPSS 20 at a 0.05 level of significance. To draw percentages of viability and CFA, the curves were graphed using Excel 2013.

## 3 Results

**Figure 1:**
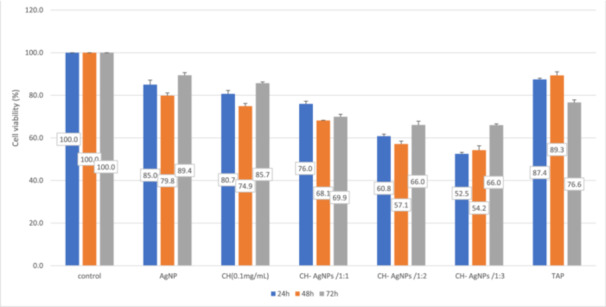
MTT assay. Cell viability after exposure to different medicaments in MC3T3‐E1 preosteoblast cell line after 24, 48, and 72 h. AgNP, silver nanoparticles; CH, calcium hydroxide; TAP, triantibiotic paste.

In this study, the negative control was arbitrarily set at 100% cell viability. Compared with the negative control, exposure to all tested medicaments decreased cell viability (Figure 1).

Considering the significant interaction of group and time, each group was evaluated at three time points, and all groups were compared at each time point of 24, 48, and 72 h (subgroup analysis).

In the AgNPs, CH, and CH‐AgNPs/1:2 groups, the highest percentage of cell viability was noted at 72 h, and the lowest percentage was recorded at 48 h. The difference between 48 and 72 h was significant in the AgNPs group (*p* < 0.05).

In the CH‐AgNPs/1:1 group, the highest percentage of cell viability was recorded at 24 h, and the lowest was recorded at 48 h. The difference in cell viability was significant among 24, 48, and 72 h.

In the CH‐AgNPs/1:3 group, the cell viability percentage gradually increased from 24 to 48 and 72 h, and the difference between 24 and 72 h as well as 24 and 48 h was significant.

In the TAP group, the highest percentage of cell viability was recorded at 48 h, and the lowest was noted at 72 h. The difference among 24, 48, and 72 h was significant.

The experimental groups were compared at each time point of 24, 48, and 72 h.

At 24 h, the TAP and AgNP groups showed a higher percentage of cell viability than the other experimental groups, and the CH‐AgNPs/1:3 group (52.5%) showed the lowest cell viability. A significant difference existed between CH‐AgNPs/1:3 with AgNPs, and TAP groups in this regard (*p* < 0.001).

At 48 h, the TAP group (89.3%) showed the highest cell viability, and the CH‐AgNPs/1:3 group (54.2%) showed the lowest cell viability, and the difference in cell viability between these groups was significant (*p* < 0.001).

At 72 h, the AgNPs (89.4%) and CH (85.7%) groups showed the highest cell viability, and the lowest cell viability belonged to the CH‐AgNPs/1:3 and CH‐AgNPs/1:2 groups (66%). The difference between CH‐AgNPs/1:3 and CH‐AgNPs/1:2 with CH and AgNPs groups was significant (*p* < 0.001).

**Figure 2:**
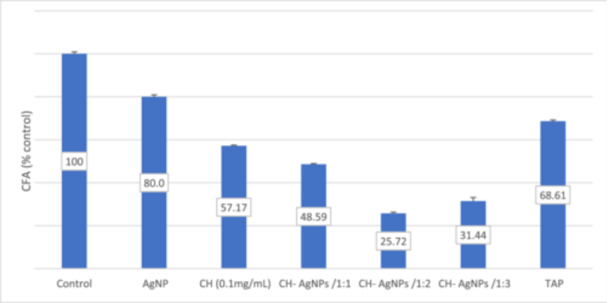
Percentage CFA of MC3T3‐E1 preosteoblast cell line related to the control after 7 days of incubation. AgNPs, silver nanoparticle; CH, calcium hydroxide; TAP, triple ntibiotic paste.

The results regarding the viability by MTT and CFA are shown in Figures 1 and 2, respectively.

## 4 Discussion

Elimination of bacteria from the root canal system is imperative for subsequent pulpal and periapical healing (Sato et al. 1993). However, minimal or no mechanical debridement is often performed in immature permanent teeth in REPs. Because of the large canal space and thin dentinal walls in such teeth, disinfection is mostly performed chemically by the use of irrigants and intracanal medicaments. An ideal medicament should be bactericidal and have broad‐spectrum antibacterial activity with no or insignificant effect on the viability, proliferation, and differentiation of stem cells, especially SCAPs (Latham et al. 2016; Verma et al. 2017; Khoshkhounejad et al. 2019; Selis et al. 2019). MC3T3‐E1 preosteoblast cells are among the various stem cells used in experiments. Several studies have used MC3T3‐E1 cells for the MTT cytotoxicity assay (Lee et al. 2007; Suh et al. 2013). In the present study, MC3T3‐E1 preosteoblast stem cells were used to assess the cytotoxicity of different root canal medicaments. Since intracanal medicaments in regeneration treatments could have cytotoxic effects on cells beyond the apex that possess the ability of hard tissue formation, in the present study, the MC3T3‐E1 cell line was chosen as the representative of hard tissue production to simulate the factual situations of a root canal system (Imazato et al. 2006).

Cytotoxicity is defined as the ability of a material to affect cell viability and is measured with different physiological indices such as reduction in cell growth and proliferation, necrosis, apoptosis, or a combination of them (Peters 2013; Labban et al. 2014). In the present study, cell viability was assessed using the MTT assay. The Trypan blue exclusive assay or the MTT assay is a colorimetric assay to assess cell viability. OD indicates the absorbance of the colored solution at a certain wavelength, and the result indicates the percentage of viable cells and the cytotoxicity of the tested material. According to the ISO guidelines, a cell viability percentage below 70% of the control group shows the cytotoxic potential (Selis et al. 2019) of the examined material (Iso B & STANDARD B. 2009) (Labban et al. 2014; Dianat, Azadnia, and Mozayeni 2015). Pintor et al. (2020) compared the MTT assay with other cell viability assays for assessment of the biocompatibility of root canal filling materials in their systematic review. They concluded that MTT and 2,3‐bis(2‐methoxy‐4‐nitro‐5‐sulfophenyl)‐5‐[(phenylamino)carbonyl]‐2H‐tetrazolium hydroxide are reliable assays and do not over‐estimate or underestimate the cell viability in cytotoxicity assessment of intracanal medicaments. Thus, they can be reliably used to assess the toxicity of various intracanal medicaments as well as their antibacterial efficacy (Latham et al. 2016; Khoshkhounejad et al. 2019). Therefore, as we investigated the antibacterial efficacy of AgNPs in our previous studies (Javidi et al. 2014; Afkhami et al. 2015), it was imperative to assess their cytotoxicity for stem cells as well.

The most popular intracanal medicaments in REPs include CH and TAP. Recent studies confirmed the safety of CH as an intracanal medicament, irrespective of its concentration or dilution, for apical and preapical stem cells (Kim et al. 2018; Gougousis et al. 2019; da Silva et al. 2020; Jain et al. 2020). Ruparel et al. (2012) reported that CH is nontoxic at all concentrations and stimulates the proliferation of SCAPs at certain concentrations. Another in vitro study demonstrated that CH had low cytotoxicity at all experimental time points and dilutions. Their results agreed with previous studies that suggested CH as a nontoxic substance compared with other medicaments such as TAP (Mizuno and Banzai 2008; Desai and Chandler 2009; Althumairy, Teixeira, and Diogenes 2014).

TAP is an effective antibacterial medicament and disinfectant for teeth with immature roots (Sato et al. 1993; Hoshino et al. 1996; Windley et al. 2005; Selis et al. 2019). Khoshkhounejad et al. (2019) demonstrated that TAP promoted the proliferation of SCAPs at any concentration compared with other medicaments. However, TAP contains minocycline, which causes tooth discoloration; thus, double antibiotic paste was suggested as an alternative medicament which consists of metronidazole and ciprofloxacin (Gougousis et al. 2019; Selis et al. 2019; da Silva et al. 2020). In the present study, TAP was used as the positive control group, and also, CH was mixed with AgNPs in various ratios.

AgNPs have gained popularity because of their potential antibacterial activity (Ahari et al. 2011; Wu et al. 2014). Wu et al. (2014) reported that AgNPs used as a medicament had a more significant antibiotic efficacy compared with when used as an irrigant. In another study, the application of 0.02% AgNPs as a medicament was compared with CH and irrigation with a higher concentration of AgNPs (0.1%). It was shown that the combination of AgNPs and CH caused a significant reduction in *Enterococcus faecalis* count. AgNPs require a long enough interaction time for effective elimination of bacteria; therefore, they need to be used as a medicament rather than an irrigant (Shrestha and Kishen 2016). In agreement with previous findings, the application of AgNPs combined with CH as an antibacterial medicament during endodontic treatment was considered in the present study. On the other hand, it is essential to consider the possible toxicity of this mixture. A few studies have addressed this topic so far. Somayyeh et al. mentioned that the toxicity of AgNPs depends on their concentration (Somayyeh et al. 2011). Therefore, a combination of CH and AgNPs with different concentrations was used in the present study to compare their cytotoxicity for MC3T3‐E1 stem cells. In addition, Matoug‐Elwerfelli et al. (2021), in their review article, reported that the biocompatibility of AgNPs was concentration‐dependent, and their cytotoxicity increased in higher concentrations. In the present study, the OD values and the cell viability percentage results were close to each other. In both tests, the AgNPs and TAP groups showed the highest percentage of cell viability and the least cytotoxicity; the highest cytotoxicity belonged to the CH‐AgNPs/1:3 group as the concentration of AgNPs increased. Thus, according to the present results and those of previous studies, it may be concluded that AgNPs have concentration‐dependent cytotoxicity.

## 5 Conclusion

AgNPs showed the least cytotoxic effects in all periods. The addition of AgNPs to CH increases the cytotoxic effects of CH on experimental cells. After 48 and 72 h, CH‐AgNPs/1:1 showed significantly higher cell viability in comparison to higher concentrations.

## Author Contributions

**Farzaneh Afhkami:** conceptualization, methodology, investigation, writing – original draft preparation, writing – review and editing. **Paniz Ahmadi:** data curation, writing – original draft preparation, writing – review and editing. **Golriz Rostami:** writing – original draft preparation, writing – review and editing.

## Ethics Statement

The authors have nothing to report.

## Consent

The authors have nothing to report.

## Conflicts of Interest

The authors declare no conflicts of interest.

## Data Availability

Data that support the findings of this study are available from the corresponding author upon reasonable request.
